# Interpretation of SARS-CoV-2 behaviour on different substrates and denaturation of virions using ethanol: an atomic force microscopy study

**DOI:** 10.1039/d0ra09083b

**Published:** 2020-12-14

**Authors:** Umit Celik, Kubra Celik, Suleyman Celik, Hasan Abayli, Kezban Can Sahna, Şükrü Tonbak, Zulal Asci Toraman, Ahmet Oral

**Affiliations:** School of Civil Aviation, Firat University Elazig 23119 Turkey u.celik@firat.edu.tr; Sabanci University Nanotechnology and Applications Center (SUNUM), Sabanci University Tuzla Istanbul 34956 Turkey; Department of Virology, Faculty of Veterinary Medicine, Firat University Elazig 23119 Turkey; Department of Microbiology, Faculty of Medicine, Firat University Elazig 23119 Turkey; Department of Physics, Middle East Technical University Çankaya Ankara 06800 Turkey; NanoMagnetics Instruments Ltd Ankara Turkey

## Abstract

Coronavirus (SARS-CoV-2) is a respiratory infection virus that was first detected in Wuhan, China. The virus causes COVID-19 disease and the outbreak was recognised as a pandemic by the World Health Organization (WHO) in March 2020. SARS-CoV-2 virion was first imaged using cryo-electron microscopy by the Chinese Center for Disease Control and Prevention (CDC). Atomic Force Microscopy is a unique technique that can allow imaging of biomolecules under different conditions. In this work, we used Atomic Force Microscopy to characterize SARS-CoV-2 on tissue culture polystyrene (TCPS) and glass coverslip surfaces. We isolated SARS-CoV-2 and drop casted it on coverslip glass and tissue culture polystyrene surfaces. We analyzed height profiles, density, and aggregation behavior of the virion on glass and polystyrene surfaces. We observed the coffee ring effect on the drop casted samples and close packing of virions near the coffee rings on both surfaces with relatively higher virion distribution on the tissue culture polystyrene (TCPS) substrates. We compare virion agglomeration on the two types of surfaces. Finally, we applied ethanol disinfectant to virions on the surface to visualize the effect of ethanol and image the ultrastructure of SARS-CoV-2.

## Abstract 2

We studied the density and aggregation behavior of virions on TCPS and glass substrates, surface functionality, coffee ring effect on both surfaces and the effect of ethanol-based disinfectant on the virion structure using atomic force microscopy.

## Graphical abstract

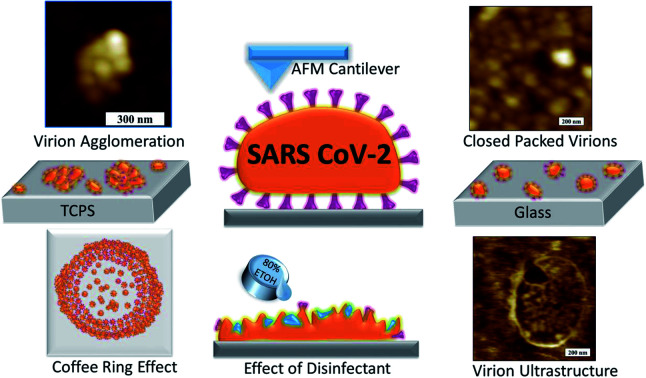

## 1. Introduction

COVID-19 is an infectious disease caused by Severe Acute Respiratory Syndrome Coronavirus-2 (SARS-CoV-2).^2^ SARS-CoV-2 has characteristics of such wide contagion and quick propagation velocity that the World Health Organization (WHO) declared the COVID-19 outbreak a global pandemic in March 2020.^3^ By November 9, 2020, the number of registered COVID-19 cases reached 50 million giving rise to a global public health emergency.^4^ In addition to the devastating loss of human life and permanent damage to health and wellbeing of millions who have survived the disease, the disruption caused by the COVID-19 pandemic has had destructive impacts on global macroeconomics,^5^ global tourism,^6^ global mobility,^7,8^ economies,^9^ the stock market,^10^ global poverty,^11^ and global development,^12^ and psychological impacts,^13–15^ lockdown effects,^16,17^ home confinement effects,^18^ environmental effects^19^ and many other effects on human wellbeing and daily living. While virologists and other health professional desperately seek to develop a vaccine for the virus, understanding the three dimensional structure of the virus has become a crucial factor in understanding how the different components of the virus interact with cells and other materials and environmental conditions. The ability to control and manipulate interactions with the virus can greatly assist us in designing procedures and products to improve public health by limiting and halting the spread of the disease.

**Fig. 1:**
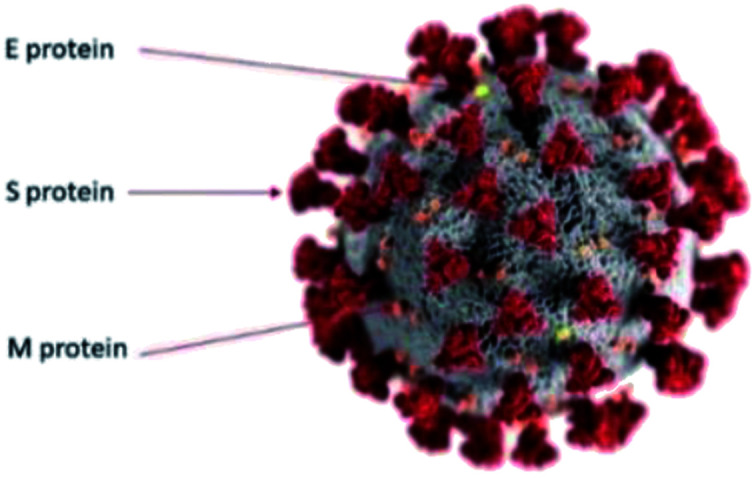
Simplified colour coded illustration model for SARS-CoV-2 virus. The gray surface is a spherical bilayer lipid envelope. The more abundant membrane or M proteins are shown in orange. The envelope or E membranes are shown in yellow and the spikey S proteins are shown in red. Image courtesy CDC/Alisa Eckert and Dan Higgins.^20^

The SARS-CoV-2 virus is the newest member belonging to corona virus family. We can utilize the popular colour-coded, illustrated visualization model of the SARS-CoV-2 virus provided by the U.S. Centers for Disease Control and Prevention (CDC) as shown in Fig. 1.^20^ The gray surface is a spherical envelope that surrounds the nucleus of the virus, containing genetic material. The grey envelope includes structural proteins called envelope (E), membrane (M) and spike (S) glycoproteins.^21^ Spike proteins (S) which are ∼20 nm in length have been shown to be extremely important for entrance of the virus into the cells.^22–24^ The diameter of the virion varies between 70 to 140 nm and average diameter is 100 nm.^25–28^ There is a review on surface behavior of corona virus family^29^ but the surface behavior of SARS-CoV-2 is different and is still under investigation. SARS-CoV-2 has been found to be more stable on plastic and stainless steel surfaces.^30^ SARS-CoV-2 has been found to be more stable on smoother surfaces,^31^ but the surface functionality perspective was missing. In another study, the stability of SARS-CoV-2 at different temperatures, surfaces and their inactivation using ethanol based and other disinfectants were investigated.^32^ The lack of any suitable molecular visualization techniques have restricted investigations at the virion/molecular level. To date, due to its miniscule size range, SARS-CoV-2 virus has been mostly observed with cryo-electron microscopy.^1,21,33,34^ However, this technique has several disadvantages *viz*, (a) the samples are usually in the form of thin slices of infected tissue and require a conductive coating (b) the imaging is performed at very low cryogenic temperatures and (c) erroneous identification of coronavirus directly in tissues have been reported^35^ and (d) the images are 2 dimensional.

Atomic Force Microscope (AFM) is a unique technique that enables imaging of biological molecules in their native environment at the molecular scale, and in some cases, has even allowed researchers to perform *in situ* experiments to observe dynamic biological activity at high resolution.^36^ In this technique, the sample do not require to be coated and the rendered images have the added advantage of being 3 dimensional. Previously, atomic force microscopy was used to characterize the ultra-structure of SARS coronavirus,^37^ and in a parallel study by Kiss *et al.*,^38^ still to be published, the authors claim to have characterized SARS-CoV-2 virus in different environments using Atomic Force Microscopy. They claim to have obtained high resolution topographical structural information and data on the thermal sensitivity and nanomechanical dynamics of SARS-CoV-2.

In this work, we isolated SARS-CoV-2 virions to investigate the topographical and phase images of the virion on tissue culture polystyrene (TCPS) and glass cover slip substrates using atomic force microscopy. We studied the density and aggregation behavior of virions on both substrates, surface functionality and coffee ring effect^39^ on both surfaces and effect of ethanol-based disinfectant on virion structure.

## 2. Materials and methods

### 2.1 SARS-CoV-2 virus isolation

Informed consents were obtained from human participants of this study. Nasopharyngeal swabs taken at an authorized diagnostic laboratory for COVID-19 from individual patients in Turkey, who were confirmed COVID-19 positive, by real-time reverse transcription polymerase chain reaction (RT-PCR) and transferred with a mixture of 10% heat-inactivated fetal bovine serum (FBS, Thermo Fisher, MA, USA), 50 U mL^−1^ penicillin, 50 μg mL^−1^ streptomycin added (Sigma-Aldrich, USA) Dulbecco's modified Eagle's medium (DMEM; Sigma-Aldrich, USA). Since the clinical samples were obtained *via* RT-PCR, only the samples showing the smallest Ct (cycle threshold) were selected for virus isolation stage. The virus isolation step was performed in a class 3 biosafety cabinet (ESCO Biotech, Singapore) in a biosafety level 3 (BSL-3) laboratory in the Virology Department, Faculty of Veterinary Medicine at Fırat University, Turkey. Following filtration through a 0.22 μm sterile membrane filter (Merck Millipore, Darmstadt, Germany), nasopharyngeal swab specimens were inoculated into African green monkey kidney (Vero E6, ATCC: CRL-1586) cells. Cells were maintained at 37 °C in 5% CO_2_ in the presence of Dulbecco's Modified Eagle's medium containing 2% fetal bovine serum, l-glutamine, 100 U mL^−1^ penicillin and 100 μg mL^−1^ streptomycin. The infected cells were monitored daily for cytopathic effect. Cytopathic effects were observed in Vero E6 cells 36 hours after inoculation and 80% of the cells were lysed at 72 hours. Before performing the centrifugation step, the cell suspension was frozen at −80 °C and thawed several times. The nuclear fraction was then separated from the lysed by centrifugation (Hettich, Germany) at 4500 rpm for 20 min at 4 °C. The virus containing supernatant was collected and stored at −80 °C as SARS-CoV-2 virus stock solution.

### 2.2 Sample preparation and AFM imaging

All sample preparations and imaging studies were carried out in a BSL3 Class Laboratory.

#### 2.2.1 Preparation of substrate surfaces

For AFM sample preparation we used cover glasses from Thermo Scientific and CELL Star tissue culture polystyrene cell culture dishes as substrates. For the polystyrene (PS) surface substrates, oxygen containing plasma treatment was performed to efficiently oxidized the surface. This treatment has been shown to enable better cell adhesion by introducing biologically relevant chemistry.^40^ Both types of substrates were rinsed with a copious amounts of Milli-Q water (18.2 MΩ cm generated from a Q-GARD 2 Milli-Q system, Millipore, Billerica, MA).

#### 2.2.2 Checking the hydrophilicity/wettability of the surfaces

A 4 μL drop of Milli-Q water was placed on surfaces to measure the water contact angle. Three different positions on the sample were tested to assure the accuracy. TCPS and glass substrate water contact angle was measured to be 49.8° and 31.9°, respectively. We measured the water contact angle of polystyrene surface without any treatment as 74.6°, confirming that the TCPS surfaces have active functional groups on their surfaces.

#### 2.2.3 Preparation of AFM samples

20 μL of purified SARS-CoV-2 solution was drop casted on to coverslip and TCPS dishes. Each sample was incubated for 15 minutes and then rinsed three times with a copious amount Milli-Q water while holding each sample about 30° angle to allow the excess water to flow evenly over the surface.

The samples were dried overnight in Petri dishes and were then inactivated by exposing the samples to UV light for 30 minutes. The samples were loaded to AFM after drying overnight.

#### 2.2.4 Imaging of AFM samples

Atomic force microscope imaging was carried out using a portable ezAFM (NanoMagnetics Instruments, U.K.) also located in a BSL3 Class laboratory. For general imaging, dynamic mode scanning was performed using Nanosensors PPP-XYNCSTR cantilevers with 7.4 N m^−1^ force constant. We used 70% amplitude setpoint and 0.5 Hz scanning rate for imaging. After completion of our experiments, the samples were bleached with 5% NaOCl solution for chemical destruction of the virus and safe disposal of samples.

#### 2.2.5 Imaging analysis AFM samples

Data was analyzed using Image Analyzer Software (NanoMagnetics Instruments, U.K.). To avoid any loss of any of finer details in the images, no filters or smoothing operations were applied to the original data. Heights of virions were measured at the center of the particles relative to the substrates.

## 3. Results and discussions

### 3.1 SARS-CoV-2 AFM topography and phase images

We imaged the dried samples on TCPS and glass substrate with the AFM in dynamic mode which provided the added advantage of phase imaging. Phase images in AFM refers to the phase lag between cantilever driving signal (at the back of cantilever where the drive piezo is located) and the signal from cantilever oscillation at the tip (where the cantilever tip interacts with the surface). Phase lag reflects the changes in mechanical properties of the surface as qualitative data.

**Fig. 2:**
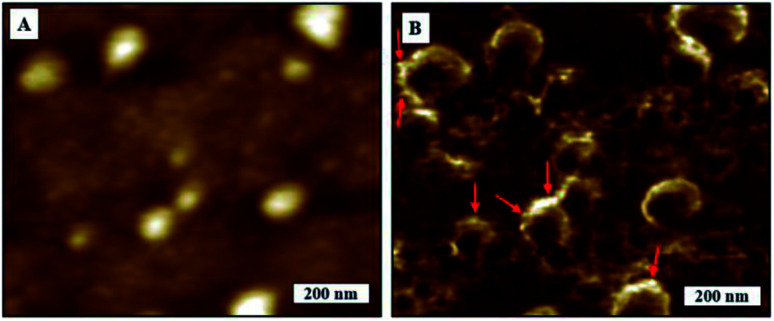
AFM images of SARS-CoV-2. (A) Topography image, (B) corresponding phase image.

We obtained high resolution SARS-CoV-2 phase images that could be linked to qualitative local mechanics of virions and corresponding topography image on TCPS substrates as in Fig. 2B and A. The globular shape of the virions are more well defined in the phase images making it easier to obtain more accurate measurements as compared to topography. We noticed a phase contrast gradient from top to bottom on the phase image which is most likely to be a tip effect. We observed some local phase contrast differences as marked on phase image at the boundaries that would corresponds to membrane proteins of SARS-CoV-2 as in Fig. 2B. The topography image is blurred possibly due to the soft samples being deformed by tip but not only is the spherical shape more visible with greater clarity in the phase image, we can also see some features on the edges and surface the virions. We observed some spiky features protruding along the edges of the virions that look like S protein but we cannot conclusively say any brighter features comes from M, E or that impurities can be ruled out completely. All we can say is that the contrast is coming from changes of interaction from differences in material properties. The features on the borders of virions are consistent in forward and backward phase images. We were unable to resolve individual virus membrane proteins on topography or phase images in spite of the radius of curvature of the cantilever tips being <7 nm. We therefore assume that the drying process performed before imaging could have denatured the surface proteins. We would require studies on higher resolution images and perhaps nanomechanical measurements to ascertain if individual proteins can be identified with this technique.

### 3.2 SARS-CoV-2 height measurements on TCPS and glass substrates

We had to adopt different approach for height measurements of the virions. One, which took into account (a) the possibility of compression of the low modulus of the virions by the AFM tip as it scanned across the surface and (b) the regions surrounding the virions may have a layer of residual products from sample preparation process.

The force applied by the AFM tip on the sample in dynamic mode scanning was calculated to be 0.2 nN by using a method proposed by Zhong *et al.*^41^ Although this is considerably smaller than the force applied in contact mode AFM there is still a possibility of some compression of the low-modulus virions by the AFM tip as it scanned across if we consider force–distances curves captured by Kiss *et al*.^38^ The height differences from middle top of the virion to the base were reported to range between 9 nm to 15 nm for glass substrate and 14 nm to 38 nm for TCPS substrate. These values are much smaller than diameter of SARS-CoV-2 virus, which were to vary from 80 to 120 nm.^25^ It therefore possible that (a) the drying of samples, causes the virions to flop flatter on to the surface and (b) the surface in the areas surrounding the virions could be coated with a by-product layer of intracellular content like proteins that are smaller than the virions. In order confirm the existence of such a by-product layer and obtain a more accurate measurement of the height of the virions, we employed a technique called nanoshaving, a method that was introduced by Liu *et al*.^42^

**Fig. 3:**
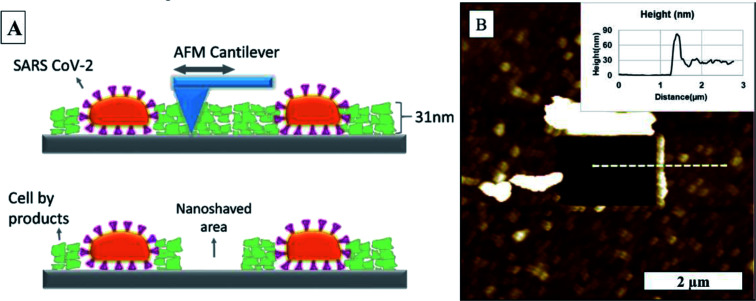
(A) Illustration of nanoshaving, (B) SARS-CoV-2 AFM topography image after nanoshaving on the glass substrate.

Using the same AFM tip, we nanoshaved the sample by operating the AFM in contact mode and applying ∼250 nN force to the surface. This ensured any residues covered the substrate surface during incubation process was removed from the substrate surface as the tip scanned across the selected area in the image as illustrated in Fig. 3A. We scanned the same region in dynamic mode afterwards to determine the height difference between the substrate and the top of the by-product layer, as shown in Fig. 3B. We used the same AFM probe for nanoshaving and imaging, so it is possible that the tip got contaminated during the nanoshaving process. Although any contamination of the tip would most certainly worsen the lateral resolution of the scanned image, it would still give us a reasonable accurate measure of the height difference between nanoshaved area to the by-product layer surface. We observed 32 nm ± 4 nm residual by product coating on the surface for both, TCPS and glass substrates. This was added to the measured central height of the virion to obtain a more accurate height measurements of SARS-CoV-2.

**Fig. 4:**
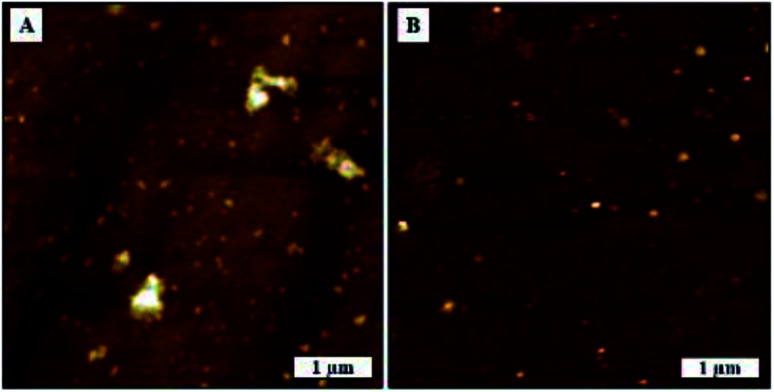
SARS-CoV-2 AFM topography images. (A) Imaged on TCPS substrate, (B) imaged on glass substrate.

**Fig. 5:**
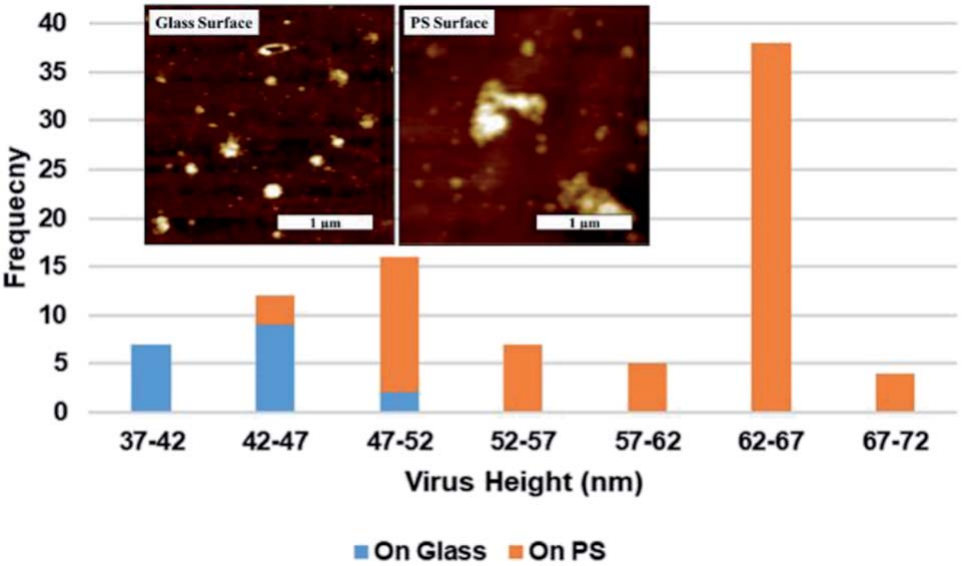
Distribution of the maximal central height of SARS-CoV-2 virions histograms on TCPS and glass substrates obtained from inset AFM topography images.

Representative AFM topography images of SARS-CoV-2 on glass substrate and TCPS substrate shown given in Fig. 4A and B respectively. Virion heights measured over a 2.5 μm^2^ area varied from 39 nm to 50 nm and 45 nm to 71 nm on the glass and TCPS surface, respectively and the height distribution for glass and TCPS surface are given in Fig. 5 along with corresponding AFM topography images showed in the insets. We manually measured heights of all virions in the AFM images according to substrate baseline. Height measurement of individual virions in aggregates can be measured higher than exact height due to stacking of virions but we measured highest individual virion height on TCPS as 66 nm which happens to be only 15 nm higher than the average virion height on glass substrate. We can therefore rule out stacking and interpret that virion on TCPS showing higher height profile because of higher relative hydrophobicity and it is much biofriendly substrate compared to glass substrate. We scanned the same samples after two months and the results was consistent those obtained from earlier experiments. We did not observe any significant changes in height differences on the samples.

### 3.3 SARS-CoV-2 density on TCPS and glass substrates

We scanned at least 10 images on the samples to obtain a measure of the virion density on glass and TCPS surfaces from their topography images. The number of virions on TCPS and glass surface in 5 μm^2^ area was counted as 153 ± 30 and 39 ± 30, respectively. The density of virions adsorbed onto TCPS surfaces was found to be ∼3.84 times higher than glass surfaces. SARS-COV-2 is an enveloped virus in which the surrounding envelope consists of a lipid bilayer that includes membrane proteins that are envelope (E), membrane (M) and the spike (S).^21^ We think active groups on the TCPS surface interacts with SARS-CoV-2 membrane proteins to give better adhesion compared to the glass substrates.

### 3.4 SARS-CoV-2 aggregation behaviour on TCPS and glass surfaces

Virions are known to agglomerate due to changes in environmental conditions such as virus particles which are released from cells.^43^ Virus aggregation-disaggregation is a complicated system and some studies have shown that virions tend to aggregate in order to increase their effective life under different environmental conditions.^44^ It is quite possible that the formation of aggregate structures of virions could lead to significant increases in the virulence transmission, as well as, prolong their effective life by acting as a protective barrier from environmental antiviral effects. It is also possible that greater aggregation of virions could be related to infecting a host cell with a higher virus load.

**Fig. 6:**
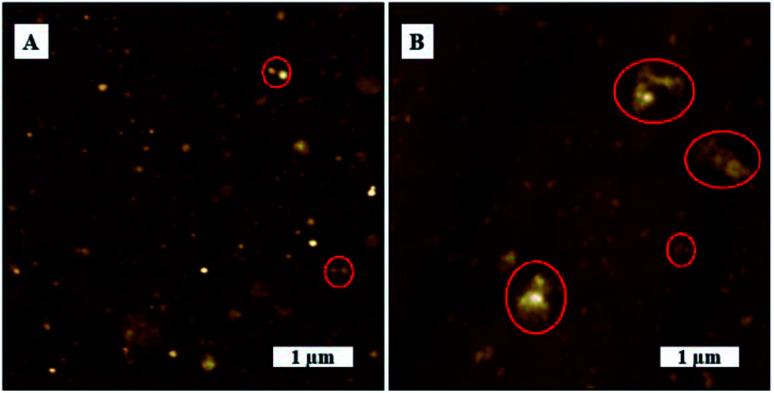
Agglomeration behaviour of SARS-CoV-2. AFM topography images, (A) on glass substrate, (B) on TCPS substrate.

In this study, we tried to investigate SARS-CoV-2 aggregation behavior by analyzing the AFM topography images of uncoated substrate surface and virion coated surfaces in greater detail. SARS-CoV-2 exhibited dense aggregation behavior on TCPS substrate showing the presence of dimers, trimers, and larger grain multimers as marked in Fig. 6B. In contrast, we observed lesser aggregation behavior on glass substrates where there were larger distributions of smaller particles and smaller dimers as marked on Fig. 6A.

**Fig. 7:**
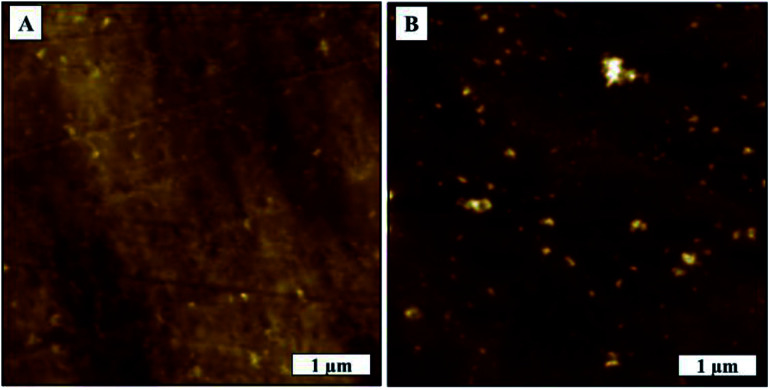
TCPS substrate AFM topography image, (A) before deposition of virion solution, (B) after deposition of virion solution.

We were able to confirm the formation of aggregated virions and eliminate surface effects by comparing the surface morphologies before and after deposition of virion solution as shown in Fig. 7A and B. We measured glass and TCPS surface average roughness as 0.67 nm and 5.06 nm, respectively. We observed rare spots with the height of 8 nm which have the same phase contrast on the TCPS surface before deposition of virion solution.

Agglomerated virions have larger mass compared to individual virions and, in the absence of any chemical interaction between surface and virions, as would be the case of the glass surface, the larger aggregates are more likely to be washed away surface during washing process. TCPS surfaces, on the other hand, have active groups on the surfaces that can interact and bind with virus membrane proteins. Consequently, the washing process is less likely to flush away the agglomerated virions from the surface. Another factor, TCPS substrate has a much rougher surface it could promote greater aggregation/cluster sites where virions get more surface area to interact/bind with the surface leading to stronger binding. This would explain why larger virion particles on TCPS surface would resist being flushed away in the washing process.

### 3.5 SARS-CoV-2 assembly enabled by the coffee ring effect

**Fig. 8:**
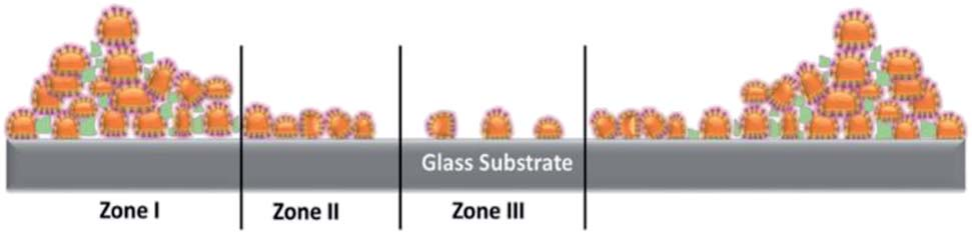
An illustration of distribution of virions on the substrates.

When it comes to deposition by drop casting, we can consider two different scenarios for the evaporation process (a) as constant contact area evaporation on hydrophilic surface and (b) as constant contact angle evaporation on hydrophobic surface. From our contact angle measurements (Section 2.2.2), both glass and TCPS surfaces, were shown to have water contact angles of less than 90°, from which we can consider both hydrophilic surfaces. Droplet evaporation mechanism on TCPS and glass substrate is based on constant contact area evaporation where evaporated on the solution-philic surface that causes coffee ring effect at the edge boundaries of droplet. An illustrative image that shows distribution of virions is given in Fig. 8.

**Fig. 9:**
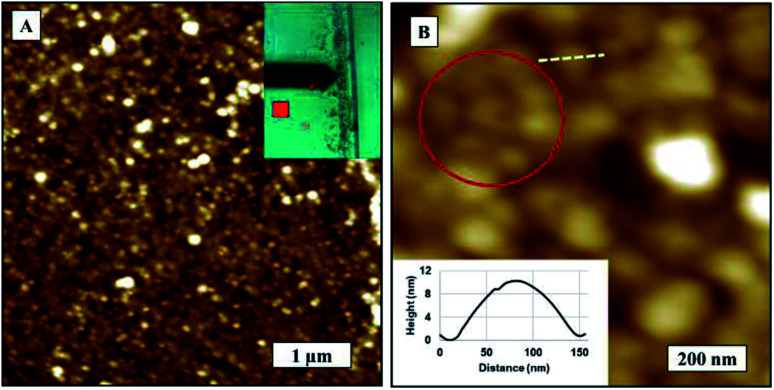
AFM images closed packed SARS-CoV-2 on glass substrate, (A) topography image, 5 × 5 μm. Inset shows the optical image of coffee ring and scanned area marked as in red. (B) Zoom in topography image of (A). Virion diameters measured as 107 ± 15 nm.

The coffee ring model is based on the fact that the evaporation rate in a droplet is higher along the edges of the droplet and the liquid from the interior is replaced by the evaporating liquid.^45^ This causes more material to accumulate at the droplet edges as illustrated in Fig. 8.^39^*Zone I* represents large agglomeration region of virions due to coffee ring effect, *Zone II* represents close packing of virions along the surface and *Zone III* represents individual and agglomerated groups of virions bound to on surface during incubation period. We observed that the evaporation of droplets shows coffee ring effect on both surfaces. TCPS substrate exhibited less material accumulated to edge of the droplets visually. However, in the *Zone III* region, the virion density is higher on TCPS surface than glass surface, this as we mentioned earlier in Section 3.2, 3.4 was probably caused by the presence of active chemical groups on the TCSP surface that interact with virus membrane proteins resulting in a more evenly distribution across the 3 zones. A stronger coffee ring effect at the droplet boundaries with closely packed SARS-CoV-2 virions near the edge boundaries (*Zone I*) on glass substrates as shown as in Fig. 9A and B. The density of virions was counted four times less in average at the droplet center on glass than on TCPS substrate. We obtained the diameter of the virus by measuring the closest neighbors center-to-center distance marked as a red circle on AFM topography image in Fig. 9B. We assume virions are spherical and the diameter of virion measured as 107 ± 15 nm. Single virion cross sectional height profile is given as an inset in Fig. 9B corresponds to dashed white line on the AFM image.

### 3.6 Exposing the SARS-CoV-2 samples to 80% ethyl alcohol disinfectant

Finally, we tried to observe the inactivation of SARS-CoV-2 virions by an ethanol-based disinfectant. Ethanol at 80% has been reported to be highly effective against SARS-CoV-2 by dissolving lipid bilayer membrane which holds the virus structure and exposing the intracellular content.^46^ In order to eliminate probability of contamination, we used ultra-pure ethanol and Milli-Q water for preparation of 80% ethyl alcohol.

**Fig. 10:**
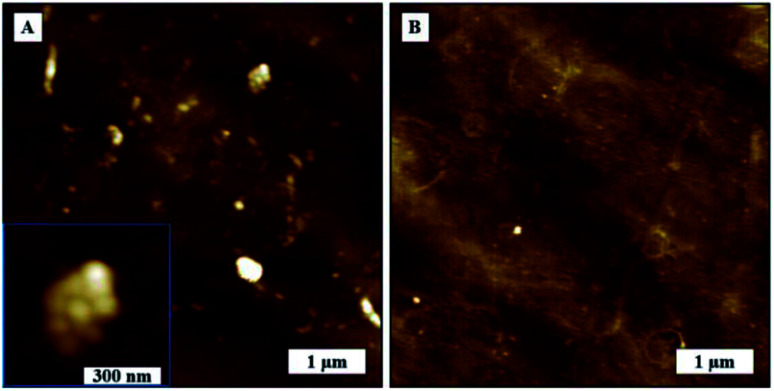
AFM topography images of SARS-CoV-2 on TCPS substrate, (A) before exposing to 80% ethyl alcohol, 5 × 5 μm^2^. Inset shows zoom in image of virus multimers. (B) Surface after exposing the specimen to 80% ethyl alcohol.

We used the same dried samples used in early experiments to drop cast a 5 μL of 80% ethyl alcohol solution onto virion surface. The sample was left to dry overnight before imaging. We observed that all globular virion structures had ruptured and flattened, leaving and virion substructures exposed on surface. SARS-CoV-2 AFM topography images on TCPS surface before and after exposing to 80% ethyl alcohol is given in Fig. 10A and B.

**Fig. 11:**
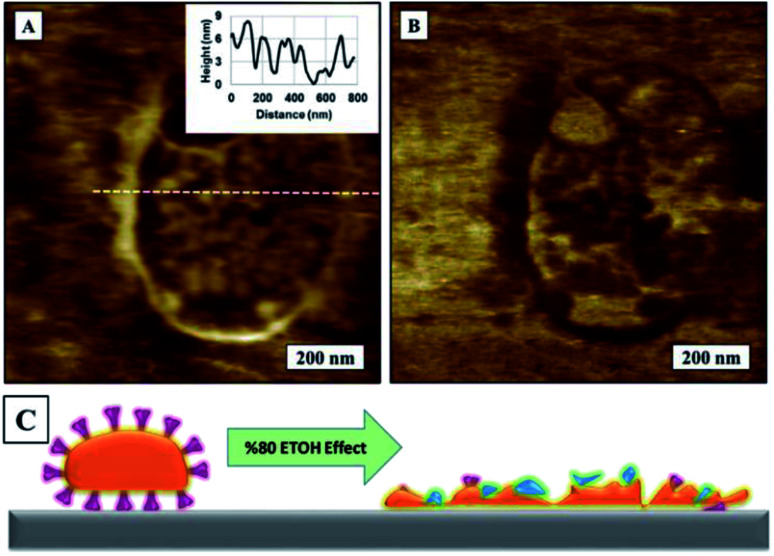
AFM images of SARS-CoV-2 on TCPS substrate, (A) topography image after exposing to 80% ethyl alcohol, (B) corresponding phase image of (A), (C) illustration of 80% ethyl alcohol effect on SARS-CoV-2.

**Fig. 12:**
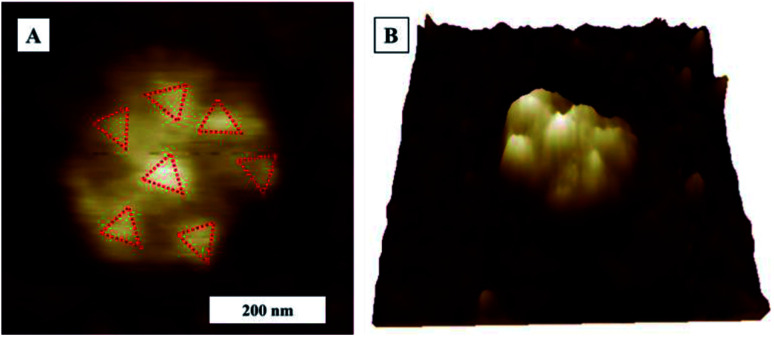
AFM Topography image of SARS-CoV-2 on glass substrate after exposing to 80% ethyl alcohol. (A) AFM topography image (B) 3D topography image of (A).

AFM topography image in Fig. 11A and corresponding AFM phase image in Fig. 11B show a zoomed close up image of a denatured virion on to surface to image of SARS-CoV-2 on TCPS following exposure to 80% ethyl alcohol. An illustration of ethanol effect on virion is given in Fig. 11C. We can confirm that 80% ethyl alcohol ruptures and inactivates the SARS-CoV-2 on TCPS surface. AFM images of what looks like spike protein array of SARS-CoV-2 on glass substrate after exposing the sample to the 80% ethyl alcohol as shown in Fig. 12. We observed the shape of the virions are deformed and flattened on glass surface after exposing 80% ethyl alcohol but it is difficult to say that they are inactivated or not.

## 4. Conclusion

In this study, we have demonstrated it is possible to effectively use atomic force microscopy to visually investigate the effects of TCPS and glass substrates on the deposition and distribution on SARS-CoV-2. We drop casted solution that included SARS-CoV-2 onto both glass and TCPS substrates without any modification. We observed virion distribution on the surface to be denser and more uniform on TCPS substrate compared to glass and recorded density and height differences on the two surfaces. We found that, due to their biofriendly surface functionality, TCPS surfaces attracted around 4 times higher virion density on the surface compared to the glass surface. Virions deposition on both surfaces showed coffee ring effect and we observed increasing virion distribution gradient from centre of the droplet to boundary edges and closely packed virion distribution near to coffee ring. We also observed that SARS-CoV-2 on TCPS substrate showed denser multimer aggregation on the surface, but we were unable to observe clear aggregation behaviour in samples cast on glass substrates. The heights of SARS-CoV-2 virions on glass and TCPS substrates were measured in range of 42 to 47 nm and 62 to 67 nm respectively. Finally, we have obtained visual confirmation that exposing the virions to 80% ethyl alcohol inactivates viral particles by rupturing their protective spherical lipid bilayer envelope.

## Conflicts of interest

There are no conflicts to declare.

## Supplementary Material
